# Development and validation of a nomogram to predict acute postoperative urinary retention in ischemic stroke patients following femoral artery puncture

**DOI:** 10.3389/fneur.2024.1435097

**Published:** 2024-10-08

**Authors:** Minfang Zhu, Weibin Zhang, Anqi Lyu, Juanbi Gao

**Affiliations:** ^1^Department of Neurology, Jiangmen Central Hospital, Jiangmen, China; ^2^Department of Pathology, Jiangmen Central Hospital, Jiangmen, China; ^3^Department of Nursing, Jiangmen Central Hospital, Jiangmen, China

**Keywords:** acute postoperative urinary retention, ischemic stroke, femoral artery puncture, nomogram, risk factors

## Abstract

**Background:**

Acute postoperative urinary retention (POUR) is a common complication in patients with ischemic stroke following femoral artery puncture (FAP), leading to discomfort, delayed hospital discharge, and increased patient morbidity. The relevant risk factors are unclear; thus, a predictive tool is required to guide treatment decisions.

**Objective:**

To develop and validate a nomogram to predict acute POUR in patients with ischemic stroke following FAP.

**Methods:**

We retrospectively collected cases from 1729 patients with ischemic stroke from the electronic record system of Jiangmen Central Hospital from January 2021 to December 2023. A total of 731 patients were randomly divided into development (*n* = 511, 70%) and validation (*n* = 220, 30%) groups. Univariate and multivariate logistic regression analyses with backward stepwise regression were used to select the predictive variables, and a nomogram was developed. The discrimination was evaluated based on the area under the curve (AUC). Calibration was assessed using calibration plots and the Hosmer–Lemeshow test. Clinical applications were evaluated using decision curve analysis (DCA).

**Results:**

The incidence of acute POUR was 12.72%. Preoperative statin use within 24 h, operation type, intraoperative infusion, postoperative water intake within 3 h, postoperative pain, and postoperative anxiety were included in the nomogram. The AUC values were 0.764 [95% confidence interval (CI): 0.705–0.825] in the development group and 0.741 (95% CI: 0.615–0.856) in the validation group. The calibration plots showed good calibration. The *p* values in the Hosmer–Lemeshow tests were 0.962 and 0.315 for the development and validation groups, respectively. The DCA showed that patients could benefit from this nomogram.

**Conclusion:**

A nomogram was developed to successfully predict acute POUR in patients with ischemic stroke following FAP. This nomogram is a convenient and effective tool for clinicians to aid in the prevention and early intervention of acute POUR.

## 1 Introduction

Stroke is a leading cause of death and disability among adults in China, posing a significant threat to the health of citizens as a major chronic disease (1, 2). According to a national stroke report (3), ischemic stroke is the most common type of stroke, accounting for 82.6% of all cases. To treat ischemic stroke, unblocking the obstructed arteries as early as possible is key, and interventional therapy with arterial puncture is necessary. Femoral artery puncture (FAP) is a relatively safe and effective interventional stroke therapy; however, FAP may lead to multiple complications, including acute postoperative urinary retention (POUR) (4).

Acute POUR is a medical emergency characterized by an abrupt inability to pass urine (5). Generally, a residual urine volume > 100 mL can be diagnosed as POUR (6), and acute POUR usually occurs within 6 h after surgery with FAP. Previous studies have reported that the incidence of acute POUR is >20% (7). Although it has been reported that stroke affecting the dominant cerebral hemisphere may lead to urinary retention (8), many patients with ischemic stroke without preoperative urinary retention experience acute POUR after surgery. Risk factors for acute POUR include age, sex, anesthesia type, puncture site, surgical duration, intraoperative infusion, and postoperative pain (9). Standard treatment with a urinary catheter is associated with the risk of infection, which can cause patient distress (10). Furthermore, acute POUR leads to discomfort, delayed hospital discharge, and increased patient morbidity (10, 11).

Clinical prediction models can integrate multiple factors into a statistical graph or scale to predict the probability of occurrence or prognosis, which can help in recommending diagnostic or therapeutic actions (12). A nomogram is a common clinical prediction model. In recent years, studies predicting POUR using nomograms have been reported. These studies have focused on various surgical procedures, such as lower limb arthroplasty (13), pelvic reconstructive surgery (14), lumbar interbody fusion surgery (15), thoracic surgery (11), and anorectal surgery (16). However, no studies have addressed interventional surgery involving FAP. In contrast, nomograms have also been used to predict urinary function indicators in ischemic stroke, such as neurogenic lower urinary tract dysfunction (17) and urinary tract infections (18). However, there are no studies on the use of nomograms for predicting acute POUR in patients with ischemic stroke. This study aimed to develop and validate an appropriate and practical nomogram for identifying the incidence of acute POUR in patients with ischemic stroke following FAP.

## 2 Methods

### 2.1 Study design and participants

**Figure 1:**
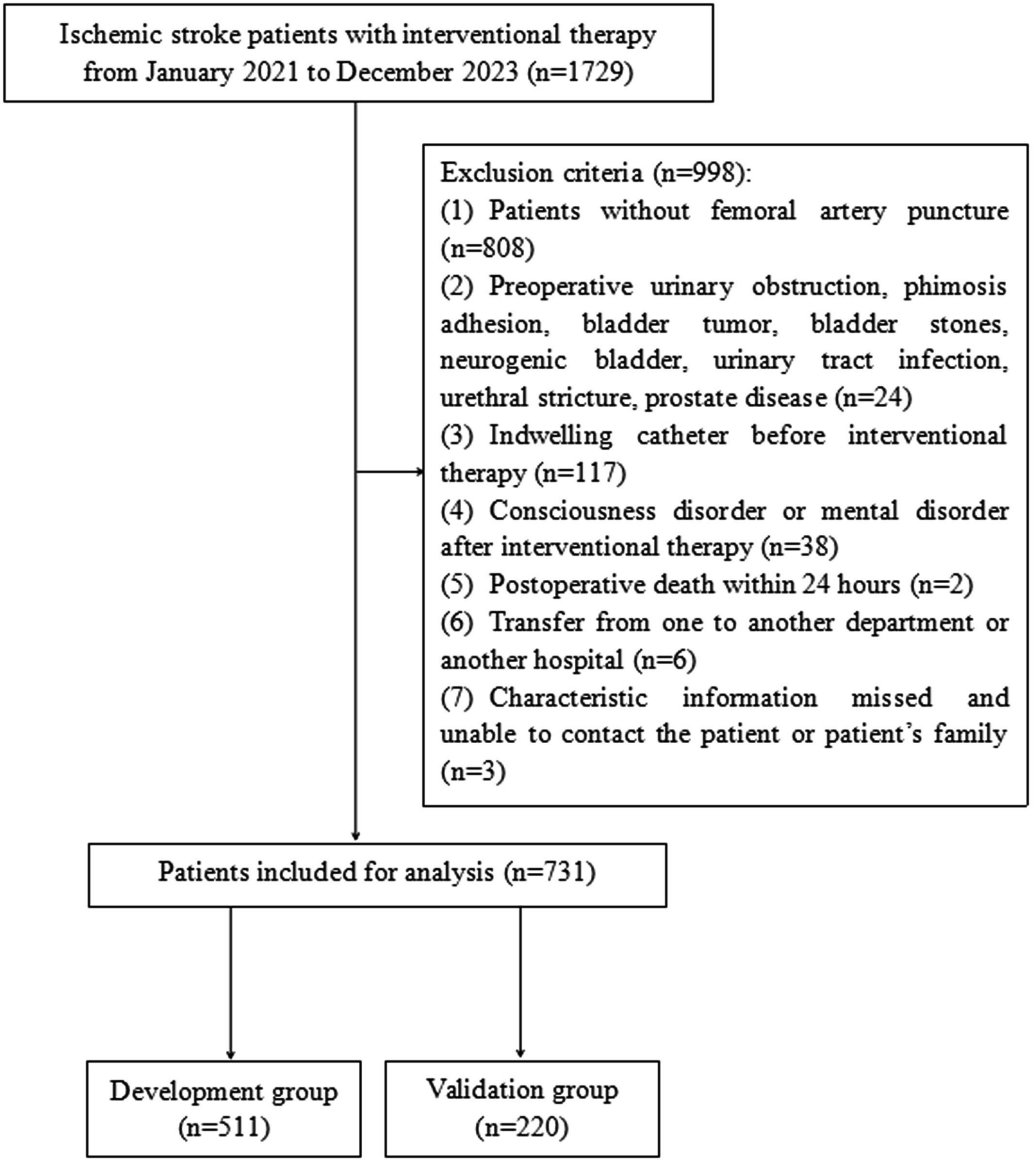
Flowchart of patient selection.

A retrospective study was performed on 1729 patients with ischemic stroke who received interventional therapy in the Neurology Department of Jiangmen Central Hospital from January 2021 to December 2023 (Figure 1). The inclusion criteria were as follows: (1) age ≥ 18 years; (2) diagnosed with ischemic stroke; and (3) completed interventional therapy during hospitalization. The exclusion criteria were as follows: (1) did not accept FAP as the interventional therapy; (2) diagnosed with diseases associated with preoperative urinary obstruction, phimosis adhesion, bladder tumor, bladder stones, neurogenic bladder, urinary tract infection, urethral stricture, or prostate disease; (3) indwelling catheter before interventional therapy; (4) consciousness disorder or mental disorder after interventional therapy; (5) postoperative death within 24 h; (6) transfer from another department or another hospital; and (7) characteristic information missed and unable to contact the patient or patient’s family. This study was reviewed and approved by the Ethics Committee of Jiangmen Central Hospital (No. 2022117).

### 2.2 Data collection

The patients’ demographic and clinical characteristics were obtained from the hospital’s electronic medical record system. Demographic characteristics included age, sex, and body mass index (BMI). Clinical characteristics included diagnosis, chronic medical history (diabetes, hypertension, coronary disease), preoperative medication within 24 h (aspirin, statin), operation type, anesthesia type, puncture site, surgical duration, intraoperative infusion, postoperative infusion, postoperative water intake within 3 h, postoperative pain, postoperative anxiety, and postoperative urination.

Acute POUR is a unique outcome indicator. If a patient cannot urinate within 6 h after surgery or if the residual urine volume after urination is >100 mL, a diagnosis of acute POUR is considered. For accurate measurements, postoperative water intake within 3 h was measured using graduated cups or syringes. Postoperative pain was assessed using a Numerical Rating Scale (NRS) (19) every hour within the 6 h period after surgery; during those 6 h, postoperative pain was diagnosed if the NRS score was 1 or higher. Postoperative anxiety was examined using the Self-Rating Anxiety Scale (SAS) at 6 h after surgery. The score was converted by multiplying the number by 1.25, with a total score of 0–100 points (20); the critical score was 50 points, with a score of 50 or above indicating postoperative anxiety (21).

### 2.3 Statistical analysis

Using the sample function in R software, the dataset was randomly divided into development (70%) and validation (30%) groups. The demographic and clinical characteristics of the two groups were compared. Continuous variables with a normal distribution were presented as mean ± standard deviation, whereas continuous variables with a non-normal distribution were reported as median and interquartile range. Categorical variables were described as numbers and percentages. Group comparisons were conducted using the Student’s *t* test for continuous variables with a normal distribution, the Mann–Whitney U test for continuous variables with a non-normal distribution, and the chi-square test was used for categorical variables.

Univariate and multivariate logistic regression analyses were conducted to select the predictive variables for the development group. Variables with a *p* < 0.05 in the univariate analysis were selected for the multivariate analysis. After backward stepwise selection, predictive variables were selected and a nomogram was developed.

Discrimination, calibration, and clinical practicality were used to evaluate the nomogram. Receiver operating characteristics (ROC) curves were drawn using the’ rms’ package, and the area under the curve (AUC) values were measured to estimate the discrimination. In general, AUC values of 0.50 represent no discrimination, 0.50–0.70 represent poor discrimination, 0.70–0.80 represent reasonable discrimination, and > 0.80 represent good discrimination (22). Calibration curves were drawn, and the Hosmer–Lemeshow test was conducted to estimate the calibration; a *p* value >0.05 indicated excellent calibration (23). Decision curve analysis (DCA) was conducted graphically to evaluate the clinical practicability.

The group division, development, and evaluation of the nomogram were performed using R software version 4.1.2 (R Project for Statistical Computing, Vienna, Austria). Other data analyses were performed using IBM SPSS version 26.0 (IBM, Armonk, NY, USA). All statistical tests were two-sided, with a *p* value <0.10 considered statistically significant in multivariate logistic regression analysis, and a *p* value <0.05 considered statistically significant in other analyses.

## 3 Results

### 3.1 Patient characteristics

A total of 1729 patients with ischemic stroke were assessed for eligibility using the hospital electronic record system between January 2021 and December 2023. We excluded 998 patients who met the exclusion criteria. A flowchart of the patient selection process is shown in Figure 1. Finally, 731 patients were included in this analysis and were randomly divided into the development (*n* = 511, 70%) and validation (*n* = 220, 30%) groups.

The demographic and clinical characteristics of all patients in the development and validation groups are presented in Table 1. Acute POUR occurred in 93 patients (12.72%): 62 patients (12.13%) in the development group and 31 patients (14.09%) in the validation group. None of the characteristics significantly differed between the development and validation groups.

**Table 1:** Baseline characteristics of all, development, and validation group.

	All patients (*n* = 731)	Development group (*n* = 511)	Validation group (*n* = 220)	*P^a^*
Age, years	63.7 ± 9.8	63.6 ± 10.0	64.2 ± 9.4	0.422
Sex, *n* (%)				
Male	487 (66.6%)	332 (65.0%)	155 (70.5%)	0.175
Female	244 (33.4%)	179 (35.0%)	65 (29.5%)	
BMI, kg/m^2^	23.8 ± 3.5	23.8 ± 3.5	23.9 ± 3.4	0.693
Diagnosis, *n* (%)				
Stroke	448 (61.3%)	313 (61.3%)	135 (61.4%)	0.755
Arterial stenosis/occlusion	243 (33.2%)	168 (32.9%)	75 (34.1%)	
Others	40 (5.5%)	30 (5.9%)	10 (4.5%)	
Diabetes, *n* (%)				
No	525 (71.8%)	360 (70.5%)	165 (75.0%)	0.244
Yes	206 (28.2%)	151 (29.5%)	55 (25.0%)	
Hypertension, *n* (%)				
No	302 (41.3%)	214 (41.9%)	88 (40.0%)	0.696
Yes	429 (58.7%)	297 (58.1%)	132 (60.0%)	
Coronary disease, *n* (%)				
No	663 (90.7%)	458 (89.6%)	205 (93.2%)	0.168
Yes	68 (9.3%)	53 (10.4%)	15 (6.8%)	
Preoperative aspirin within 24 h, *n* (%)				
No	128 (17.5%)	97 (19.0%)	31 (14.1%)	0.136
Yes	603 (82.5%)	414 (81.0%)	189 (85.9%)	
Preoperative statin within 24 h, *n* (%)				
No	77 (10.5%)	57 (11.2%)	20 (9.1%)	0.482
Yes	654 (89.5%)	454 (88.8%)	200 (90.9%)	
Operation type, *n* (%)				
Selective operation	727 (99.5%)	508 (99.4%)	219 (99.5%)	1.000
Emergency operation	4 (0.5%)	3 (0.6%)	1 (0.5%)	
Anesthesia type, *n* (%)				
Local anesthesia	730 (99.9%)	510 (99.8%)	220 (100.0%)	1.000
General anesthesia	1 (0.1%)	1 (0.2%)	0 (0.0)	
Puncture site, *n* (%)				
Left lower limb	725 (99.2%)	506 (99.0%)	219 (99.5%)	0.674
Right lower limb	6 (0.8%)	5 (1.0%)	1 (0.5%)	
Surgical duration, min	50.0 (40.0, 60.0)	50.0 (40.0, 60.0)	49.5 (40.0, 60.0)	0.087
Intraoperative infusion, mL	273.0 (228.0, 336.0)	275.0 (228.0, 342.0)	267.0 (228.5, 322.0)	0.077
Postoperative infusion, mL	750.0 (500.0, 1000.0)	750.0 (500.0, 1000.0)	750.0 (500.0, 1000.0)	0.551
Postoperative water intake within 3 h, mL	750.0 (580.0, 880.0)	750.0 (500.0, 880.0)	740.0 (580.0, 880.0)	0.825
Postoperative pain, *n* (%)				
No	622 (85.1%)	439 (85.9%)	183 (83.2%)	0.403
Yes	109 (14.9%)	72 (14.1%)	37 (16.8%)	
Postoperative anxiety, *n* (%)				
No	599 (81.9%)	418 (81.8%)	181 (82.30%)	0.946
Yes	132 (18.1%)	93 (18.2%)	39 (17.7%)	

^ap < 0.05 was considered statistically significant.^

### 3.2 Selection of predictive factors

The univariate analysis showed that seven factors, including preoperative statin use within 24 h, operation type, surgical duration, intraoperative infusion, postoperative water intake within 3 h, postoperative pain, and postoperative anxiety, were associated with acute POUR in the development group (Table 2). These factors were included in multivariate logistic regression analysis. After adopting the backward stepwise method, six factors had a significant impact on the predictive ability of acute POUR: preoperative statin use within 24 h, operation type, intraoperative infusion, postoperative water intake within 3 h, postoperative pain, and postoperative anxiety (Table 3).

**Table 2:** Univariate analysis for risks factors in the development group.

	Univariate analysis	
	OR (95% CI)	*P^a^*
Age, years	0.978 (0.955–1.002)	0.077
Sex		
Male	Reference	
Female	1.316 (0.783–2.212)	0.300
BMI, kg/m^2^	1.003 (0.934–1.078)	0.926
Diagnosis		
Stroke	Reference	
Arterial stenosis/occlusion	0.945 (0.550–1.623)	0.837
Others	0.205 (0.027–1.546)	0.124
Diabetes		
No	Reference	0.129
Yes	0.625 (0.341–1.146)	
Hypertension		
No	Reference	0.581
Yes	0.866 (0.520–1.443)	
Coronary disease		
No	Reference	0.435
Yes	1.357 (0.630–2.921)	
Preoperative aspirin within 24 h		
No	Reference	0.309
Yes	1.446 (0.711–2.941)	
Preoperative statin within 24 h		
No	Reference	0.033
Yes	4.761 (1.134–19.985)	
Operation type		
Selective operation	Reference	0.036
Emergency operation	13.164 (1.177–147.182)	
Puncture site		
Left lower limb	Reference	0.672
Right lower limb	0.621 (0.068–5.639)	
Surgical duration, min	1.011 (1.002–1.020)	0.022
Intraoperative infusion, mL	1.003 (1.001–1.005)	0.010
Postoperative infusion, mL	1.000 (0.999–1.001)	0.516
Postoperative water intake within 3 h, mL	0.998 (0.997–0.999)	<0.001
Postoperative pain		
No	Reference	<0.001
Yes	5.205 (2.930–9.248)	
Postoperative anxiety		
No	Reference	<0.001
Yes	4.628 (2.682–7.985)	

^ap < 0.05 was considered statistically significant. OR, odds ratio. CI, confidence interval.^

**Table 3:** Multivariate logistics regression analysis for risks factors in the development group.

	Multivariate logistics regression analysis	
	OR (95% CI)	*P^a^*
Preoperative statin within 24 h		
No	Reference	0.038
Yes	4.798 (1.090–21.130)	
Operation type		
Selective operation	Reference	0.065
Emergency operation	11.154 (0.865–143.882)	
Intraoperative infusion, mL	1.002 (1.000–1.005)	0.055
Postoperative water intake within 3 h, mL	0.998 (0.997–1.000)	0.011
Postoperative pain		
No	Reference	0.003
Yes	2.915 (1.432–5.935)	
Postoperative anxiety		
No	Reference	0.005
Yes	2.582 (1.322–5.045)	

^ap < 0.10 was considered statistically significant. OR, odds ratio. CI, confidence interval.^

### 3.3 Establishment and evaluation of a nomogram for predicting acute POUR

**Figure 2:**
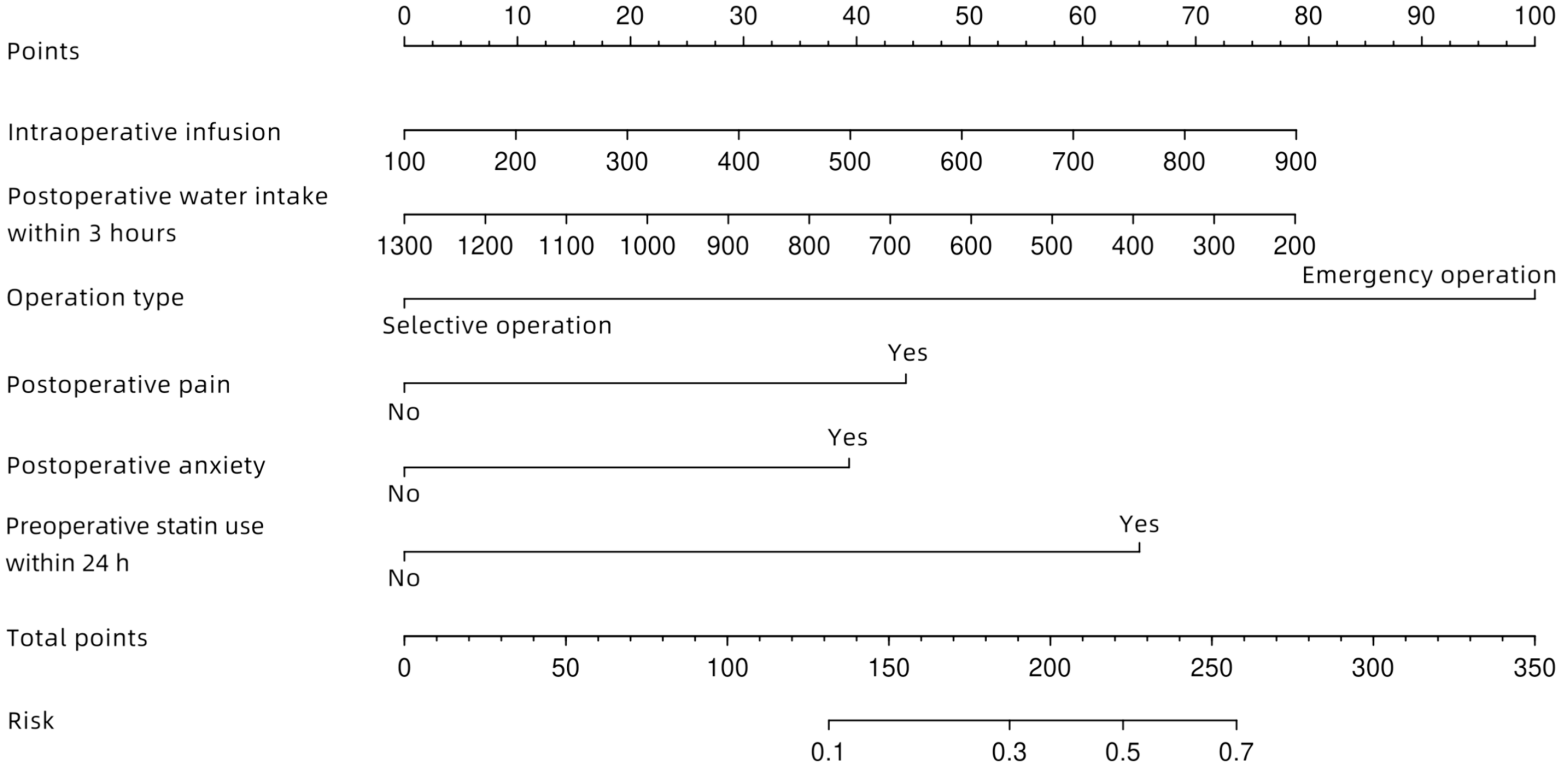
The nomogram for predicting acute POUR among ischemic stroke patients after femoral artery puncture. Rows 2 to 7 represent variables. A vertical line should be drawn from each variable to the points axis to determine the effect of each variable as a defined number of points. These points should then be summed and plotted in row 8 (total points). Finally, a vertical line should be drawn from row 8 (total points) to row 10 (risk of acute POUR) to obtain the predicted probability of acute POUR.

A nomogram for predicting acute POUR was established in the development group based on six significant factors: intraoperative infusion, postoperative water intake within 3 h, operation type, postoperative pain, postoperative anxiety, and preoperative statin use within 24 h (Figure 2).

**Figure 3:**
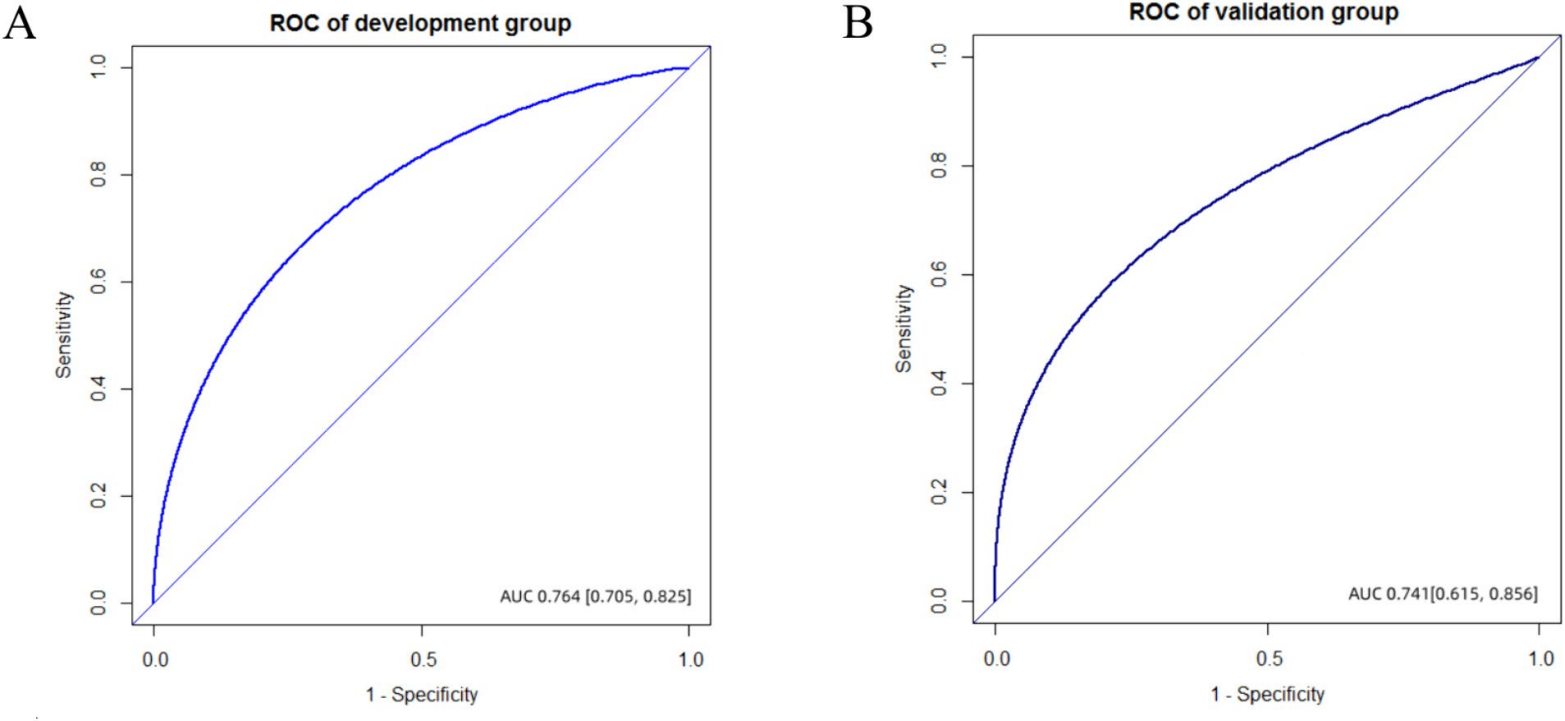
The ROC of development and validation group. **(A)** AUC value was 0.764 (95%CI, 0.705–0.825) in the development group. **(B)** AUC value was 0.741 (95%CI, 0.615–0.856) in the validation group.

**Figure 4:**
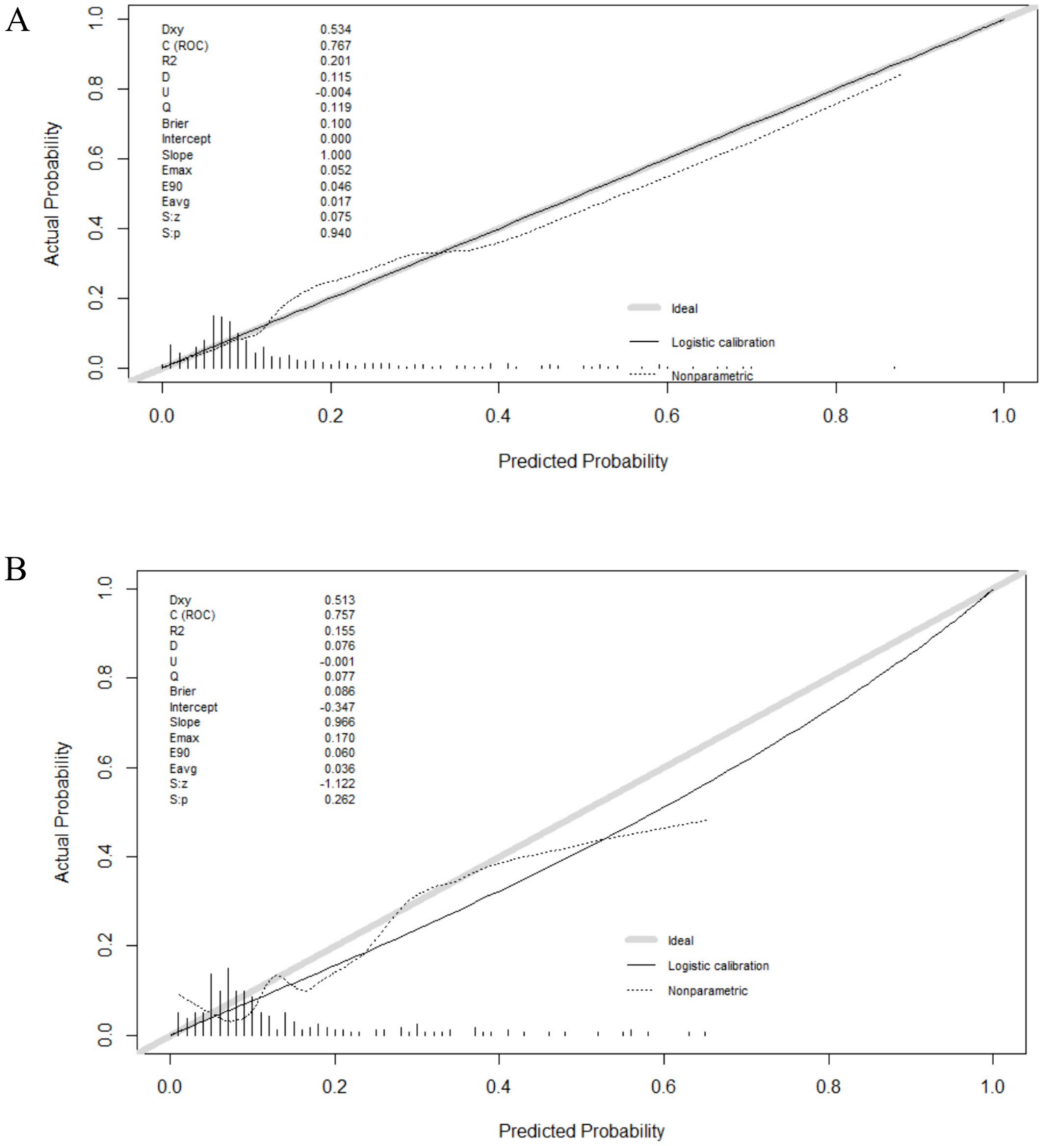
The calibration curves of the nomogram. **(A)** The calibration curve of the development group. **(B)** The calibration curve of the validation group.

Discrimination and calibration of the nomograms were evaluated. To examine the discriminative ability of the nomogram, ROC curves were plotted, and the AUC values were calculated. The AUC values were 0.764 (95% confidence interval (CI): 0.705–0.825) in the development group and 0.741 (95% CI: 0.615–0.856) in the validation group, indicating good diagnostic ability (Figure 3). Calibration of the nomogram was examined using a calibration curve and the Hosmer–Lemeshow test. As shown in Figure 4, the calibration curves indicated that the probabilities of acute POUR predicted by the nomogram were generally in accordance with the actual probabilities. The *p* values in the Hosmer–Lemeshow tests were 0.962 in the development group and 0.315 in the validation group. Comprehensively, the nomogram showed agreement between the observation and prediction.

**Figure 5:**
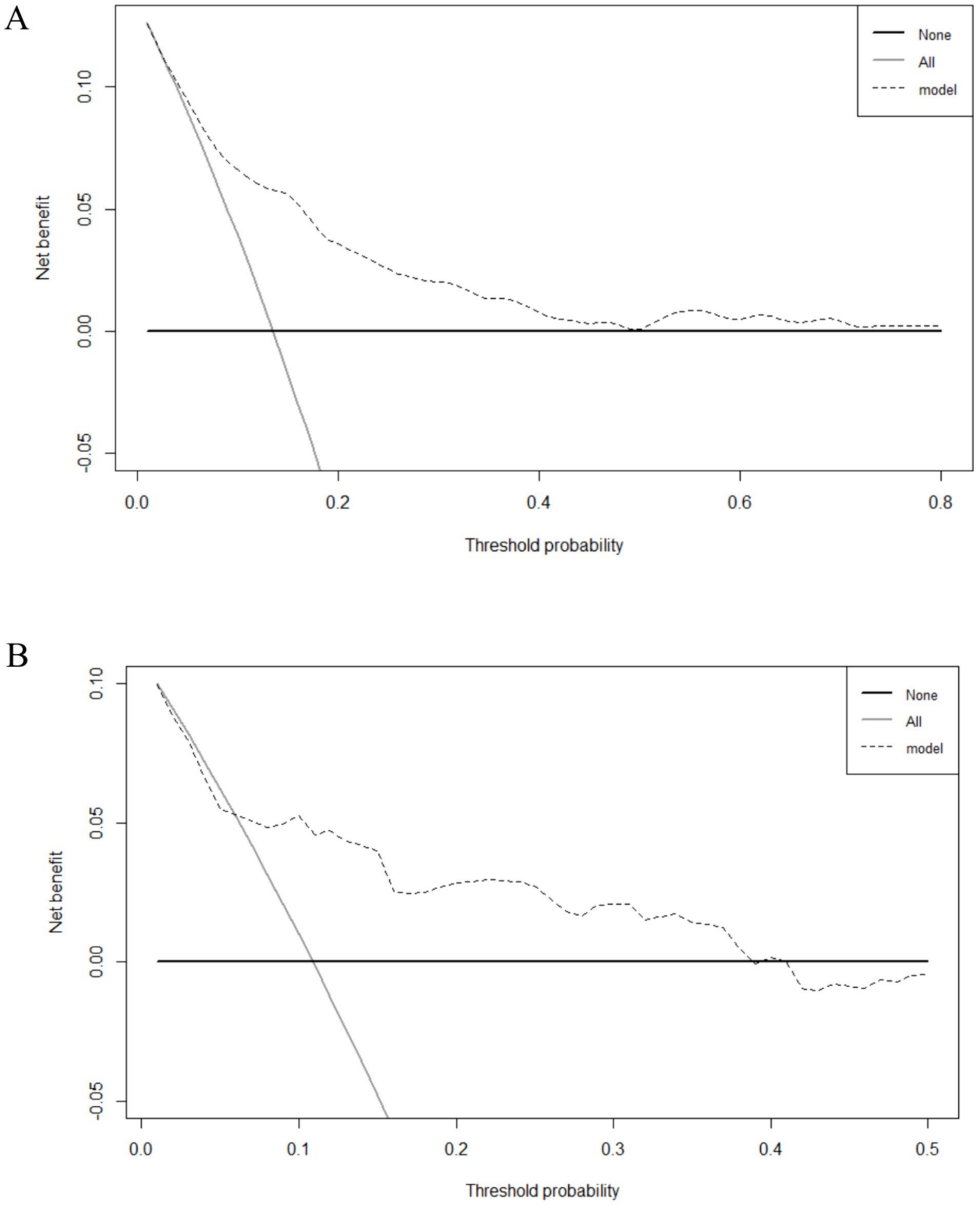
Decision curve analysis (DCA) of the nomogram. **(A)** The DCA of the development group. **(B)** The DCA of the validation group.

For clinical application, we evaluated the practicability of the nomogram using DCA. As shown in Figure 5, the threshold probability (Pt) value of the DCA in the validation group was approximately 40%, indicating that patients could benefit from this nomogram, highlighting its clinical value.

## 4 Discussion

This retrospective analysis found that the prevalence of acute POUR in patients with stroke following FAP was 12.72%, which was lower than that reported in previous studies (29–40%) (7, 24). This discrepancy could be explained by differences in subject features and observation durations. Normal bladder contraction and urine release are associated with the generation and transmission of neural information (25). Many nonsurgical features, such as bladder tumors, neurogenic bladder, and prostate disease, may negatively affect this function, leading to urinary retention. In this study, patients with these nonsurgical features were excluded, resulting in a lower incidence. A clinical consensus on the management of POUR drafted by American experts in 2023 indicated that POUR, including both acute and chronic POUR, can occur within 6 weeks after surgery (26). In our study, the observation duration for POUR was 6 h, which also resulted in a lower reported incidence.

To predict acute POUR, a diagnostic nomogram was developed based on physiological and psychological factors, including preoperative statin use, operation type, intraoperative infusion, postoperative water intake within 3 h, postoperative pain, and postoperative anxiety. These factors are easy to measure during the perioperative period of interventional therapy in patients with stroke. Strictly following the six stages indicated in a previous study (27), our nomogram showed good discrimination and calibration in the development group. Furthermore, the nomogram exhibited reasonable discrimination and calibration when applied to the validation group. Therefore, this nomogram is a convenient tool for predicting acute POUR in patients with ischemic stroke following surgery.

The factors included in our nomogram were physiological and psychological factors, most of which have been previously reported to be associated with POUR. Statins are a common medication in the lipid-lowering treatment of stroke; however, preoperative statin use can increase the risk of acute POUR, which is consistent with the findings of a previous study (28). This is because statins may damage the bladder’s smooth muscle and induce retention and an underactive bladder (29). A recent study demonstrated that excessive intraoperative infusion is a risk factor for POUR in patients undergoing colorectal surgery (9). Our study also found that intraoperative infusions were associated with a higher incidence of acute POUR. Compared to intraoperative infusion, the volume of postoperative water intake has the opposite effect on acute POUR because of the different intake pathways and durations. Generally, intraoperative infusion directly increases the fluid volume of the body through blood vessels in a short period of time, whereas postoperative water intake is accomplished through oral or nasal feeding within a reasonable period, depending on the different absorption mechanisms. Postoperative pain was significantly associated with an increased rate of acute POUR in our study, which is consistent with an American study (30). Furthermore, it is also found that stroke patients with positive postoperative anxiety have a higher incidence of acute POUR. After FAP, patients with stroke must stay in bed for at least 6 h, which can result in anxiety. Timely nursing guidance and relaxation may reduce postoperative anxiety and prevent acute POUR.

Older patients may be more prone to develop POUR (9, 31, 32); however, age was not a risk factor in our study. Many older patients were also diagnosed with preoperative urinary obstruction, bladder stones, or prostate disease; therefore, they were not included in the study.

Our study has some limitations. First, this was a single-center, retrospective study. All variables were collected through the hospital’s electronic medical record system, which is a limiting factor in the analysis. Additional variables, such as perioperative blood and imaging indicators, could be included in further cohort studies. Second, owing to limited data, the type of anesthesia was not included in the univariate analysis. Using a case–control design in a future study may help to analyze the relationships between anesthesia and acute POUR. Third, while the scores of the nomogram could be calculated, the cut-off value between high and low risk was not determined in this study. The next step should focus on the application of the nomogram and on identifying the cut-off value through the analysis of a larger dataset. Finally, the discrimination, calibration, and clinical practicability of the nomogram were excellent in the development group but reasonable in the validation group, indicating that the model could be further improved.

## 5 Conclusion

We developed and validated a nomogram to predict acute POUR following FAP in patients with ischemic stroke. This nomogram included common physiological and psychological factors and could be used as a convenient and effective tool for acute POUR prevention and intervention.

## Data Availability

The raw data supporting the conclusions of this article will be made available by the authors, without undue reservation.
